# Flexible and Stretchable Bioelectronics

**DOI:** 10.3390/ma15051664

**Published:** 2022-02-23

**Authors:** Chandani Chitrakar, Eric Hedrick, Lauren Adegoke, Melanie Ecker

**Affiliations:** Department of Biomedical Engineering, University of North Texas, Denton, TX 76203, USA; chandanichitrakar@my.unt.edu (C.C.); erichedrick@my.unt.edu (E.H.); laurenadegoke@my.unt.edu (L.A.)

**Keywords:** flexible and stretchable bioelectronics, stretchable polymer, conductive polymers, stretchable sensors, flexible and stretchable power sources, stretchable batteries, supercapacitors, fabrication of stretchable bioelectronics

## Abstract

Medical science technology has improved tremendously over the decades with the invention of robotic surgery, gene editing, immune therapy, etc. However, scientists are now recognizing the significance of ‘biological circuits’ i.e., bodily innate electrical systems for the healthy functioning of the body or for any disease conditions. Therefore, the current trend in the medical field is to understand the role of these biological circuits and exploit their advantages for therapeutic purposes. Bioelectronics, devised with these aims, work by resetting, stimulating, or blocking the electrical pathways. Bioelectronics are also used to monitor the biological cues to assess the homeostasis of the body. In a way, they bridge the gap between drug-based interventions and medical devices. With this in mind, scientists are now working towards developing flexible and stretchable miniaturized bioelectronics that can easily conform to the tissue topology, are non-toxic, elicit no immune reaction, and address the issues that drugs are unable to solve. Since the bioelectronic devices that come in contact with the body or body organs need to establish an unobstructed interface with the respective site, it is crucial that those bioelectronics are not only flexible but also stretchable for constant monitoring of the biological signals. Understanding the challenges of fabricating soft stretchable devices, we review several flexible and stretchable materials used as substrate, stretchable electrical conduits and encapsulation, design modifications for stretchability, fabrication techniques, methods of signal transmission and monitoring, and the power sources for these stretchable bioelectronics. Ultimately, these bioelectronic devices can be used for wide range of applications from skin bioelectronics and biosensing devices, to neural implants for diagnostic or therapeutic purposes.

## 1. Introduction

The field of bioelectronics, which goes by the name of neuromodulation, bio-stimulation, electroceuticals, wearables, implantables, etc., is an emerging field either as an alternative or as an add-on to chemical and biologic drugs [1]. With pacemakers and deep-brain stimulators, the idea of bioelectronics is not new [1]. Bioelectronics can be miniaturized to micro and nano scales with the help of Micro-Electro-Mechanical-Systems (MEMS) and nanotechnology [2], without compromising the efficacy of the devices. They can also be designed to elicit a low level of inflammatory response. Although a great improvement in miniaturized, precise, and accurate bioelectronic devices has been achieved, the next generation of bioelectronics (implantable or wearable) will require incorporating mechanical flexibility and stretchability into the device for the improved mechanical compliance between the tissue and the implanted device, and to minimize the foreign body response that limits the lifetime of these devices [3].

With the understanding that the human body functions and communicates through biophysical cues such as electrical, thermal, mechanical, and topographic signals [4], tremendous advances in developing tools have been achieved to sense and acquire those physiological signals for diagnostic purposes or to introduce physical stimuli for preventative and therapeutic purposes. Those tools/devices utilize substrates or an encapsulation layer, semiconductors as a functional interface with the biological material, and a power supply [5]. Conventional metal and silicon-based devices are bulky and rigid [6]. They are not applicable as wearable devices and as implantable devices. On the contrary, those inorganic materials offer the advantages of having high charge carrier mobilities and help in signal transduction mechanisms at the bio-interface, thereby enabling a fast response with high sensitivity [4]. Hence, significant interest lies in an emerging field of flexible and stretchable bioelectronics to integrate with the soft and complicated surface topography of the biological tissue. Flexible/stretchable bioelectronic devices are defined as those devices that can bend and undergo mechanical deformation with the ability to conform to biological tissue while maintaining electrical integrity under deformation [7]. In addition, the flexible/stretchable bioelectronic devices should be stable, biodegradable, and biocompatible. Several measures have been explored to add the flexibility and stretchability properties to biomedical devices, ranging from using elastomeric polymers as substrates and organic materials as electrodes, to providing a specific stretchable architecture to the inorganic conductor materials [8]. Engineering flexible and stretchable bioelectronics has a broad field of biomedical applications such as monitoring electrophysiological (electroencephalogram (EEG), electrocardiogram (ECG)), physiological (temperature, heart rate, etc.), mechanical (strain, blood pressure, etc.), and biochemical (glucose level, enzyme level, etc.) signals [9]. In this paper, we provide a complete review from the fabrication perspective by summarizing the (i) materials for substrates and stretchable electrodes; (ii) structural design and fabrication methods to adapt to large deformations without considerable damage to the device and biological structure; (iii) methods of signal transduction and communication between the stretchable device and recording device; and (iv) power sources for the operation of the fabricated stretchable devices.

## 2. Opportunities and Limitations in Bioelectronic Devices

Medical science has progressed tremendously in recent years. The previous gold standards in medical science were therapeutic drugs for the treatment of diseases or disorders, and imaging, blood testing, biopsies, etc. for diagnostics. With the increase in research in gene therapy, gene silencing via CRISPR, and siRNA, gene editing has shifted the theme of medical science from the earlier gold standards to these technologies. However, the bioelectronic approaches for delivering therapeutic benefits are gradually being recognized because the electrical systems of the body contribute to the healthy functioning of the body. The potential of bioelectronics to be used in bodily conditions controlled by biological circuits is one of the aspects that make it a competitor to the conventional method of using drugs. Another advantage is that these bioelectronic devices can facilitate precise treatment, meaning the therapeutic effect can be delivered locally only to the part that needs the treatment. In contrast, drugs affect the whole body, which often raises the issue of harmful side effects. Bioelectronics can also be utilized to realize smart drug delivery systems for localized targeted delivery to reduce the side effects of the drugs. Bioelectronics have also been implemented to stimulate nerves and modulate the neuronal signals for treating diseases or disorders such as drug-resistant epilepsy [10], rheumatoid arthritis [11], treatment-resistant depression [12], and neuropathic pain [13].

Bioelectronic devices are devices that use electricity to regulate biological processes, treat diseases, or restore lost functionality [14]. They can be classified into wearable or implantable devices depending on their placement. Skin mountable bioelectronics devices have gained significant traction due to their non-invasiveness in continual monitoring of physiological signals or for minimally-invasive localized drug delivery. Some of the examples of skin mountable bioelectronics include: (i) skin-mountable strain or pressure sensors for monitoring various human motions and physiological parameters [15]; (ii) electrophysiological sensors for electromyography (EMG) and electroencephalography (ECG) for the diagnosis of diseases such as epilepsy, dysphasia, and dementia; (iii) electrochemical and microneedle-based biosensors that allow noninvasive and continuous monitoring of biomarkers [16,17]; and (iv) smart theranostic drug delivery systems, such as smart contact lenses for diabetic diagnosis and therapy [18].

Bioelectronic devices also include devices that work on the principle of neuromodulation, which modify neural signals via deep-brain stimulation, vagus nerve stimulation, spinal nerve stimulation, and retinal and auditory implants [19]. Koopman et al. reported that vagus nerve stimulation (VNS) inhibits tumor necrosis factor, an inflammatory molecule that is a major therapeutic target in rheumatoid arthritis (RA), and which attenuates disease severity [20]. FDA Investigational Device Exemption (IDE) for the novel bioelectronic device to treat RA has allowed the device to be used in a clinical trial to collect safety and effectiveness data [11]. VNS has been established as a treatment method for drug-resistant epilepsy to reduce the number, length, and severity of seizures. Similarly, VNS has shown its efficacy for treatment-resistant depression [21]. Deep-brain stimulation has become a routine treatment for disorders such as Parkinson’s disease, tremor, epilepsy, and obsessive-compulsive disorder for patients whose symptoms are not treated with medications. Recently, a pioneering “brain-to-spine” wireless implant, also known as a stimulation movement overground (STIMO) device, has enabled paraplegic patients to walk with the help of crutches or a walker [22]. All these stimulation treatment methods utilize a pacemaker-like device that is implanted on the chest or abdomen area. A wire connected to the device is then routed to the electrodes placed in areas of brain, nerve, or spinal cord. The implanted electrodes produce electrical impulses that modulate the abnormal impulses to alleviate the symptoms associated with the disorders. Stimulation parameters such as frequency, amplitude, pulse width, and duration are adjusted to maximize the therapeutic effect while minimizing the side effects for facilitating individualized neuromodulation therapy [23]. Retinal implants (Argus II Retinal prosthesis system, Second Sight medical Technology) have enabled patients to regain some lost vision. These devices consist of a receiver, transmitting coil, and an electrode array that stimulate the retina to induce vision in patients with retinal degeneration [24]. Auditory implant types, such as cochlear implants and auditory brainstem implants (ABIs), have a neuromodulatory function [19]. Cochlear implants stimulate auditory nerve fibers via electrodes implanted in the cochlea to help patients with sensorineural hearing loss using functional auditory nerve fibers. ABIs stimulate the hearing pathways in the brain stem, bypassing the inner ear and auditory nerve to help patients with missing or small auditory nerves [25]. One of the breakthroughs in the field of bioelectronics is the development of the first bioelectronic implantable device that stimulates nerve regeneration by the application of electrical pulses. Following the healing of the nerve, the device disintegrates and disappears without any harmful side effects [26].

Following the above introduction to promising diagnostic and therapeutic bioelectronic devices, we now discuss several challenges that exist when fabricating or using bioelectronic devices. First, a challenge exists in creating better sensors to increase bandwidth [27] and in the development of novel fabrication techniques. Second, maintaining long-term biocompatibility, reliability, and stability of bioelectronic devices in vivo for chronic application are key challenges. For skin-mounted devices or implantable devices, it is also critical to maintain long-term conformability between the device and the elastic, wrinkled skin, or tissue. The stability of bioelectronic devices can deteriorate due to corrosion in the metal electrodes upon contact with biological electrolytes, susceptibility of hydrolysis and oxidation to specific bonds in polymer structures, and delamination providing access to deeper structures with the disruption in insulation barriers [28]. This delamination due to disruption in insulation barriers or due to repetitive stretching of the skin or tissue may cause a lack of conformability, which increases the impedances and decreases the signal-to-noise ratio (SNR). Miniaturized ultra-small implantable technology, such as “neural dust” [29] and “Stim dust” [30], enables access to small target areas and can be implanted anywhere in the body, and interfaces directly with the tissue or organ. Miniaturized biosensors also require small volumes for biological assays. However, with miniaturization comes the issue of optimizing the configuration of electrodes and compromising the available power due to the need to reduce the battery size for miniaturization [31]. In addition to these challenges, a key issue is the mechanical mismatch, particularly between the bioelectronic device and the biological tissue or organ that it contacts. The mechanical mismatch arises due to the material properties or the geometry of the device. Conventional metal or silicon-based bioelectronics are stiff. As a result, they are not only incapable of making tight contact with the tissue, but also cause irritation to the tissue. Therefore, it is critical to fabricate a bioelectronic device that has mechanical compliance with the tissue. This means the device needs to be soft to adapt to the complicated surface curvature of tissue, and stretchable to comply with the repetitive stretching of the skin or tissue (such as muscle tissue, gut tissue, blood vessel, and ducts). Various fabrication techniques have been established for fabrication of hard and stiff bioelectronic devices, which can be used as a foundation to develop soft and stretchable bioelectronics. However, advances in fabrication technologies are required to potentially solve the issues associated with the fabrication of soft, stretchable bioelectronics. Hence, the main focus of this paper is the realization of soft and stretchable bioelectronics, by reviewing recent advances in materials, fabrication technologies for sensors and transducers, communication methods, and power sources.

## 3. Materials

In general, the ideal materials for bioelectronics are nontoxic, prevent direct harm to the body, and minimize the initiation of the immune response. For wearable and implantable devices, biocompatibility requirements differ. Table 1 compares the different biocompatibility requirements between the two categories of bioelectronics.

Bioelectronics that interact directly or indirectly with blood must be hemocompatible; that is, the device must prevent hemolysis, or the lysis of red blood cells. Both types of devices must be nontoxic to cells. For wearables, cytotoxicity can occur when substances leach into the skin. With implanted devices, cell damage can be caused by leaking substances, and physically by the device. Wearable bioelectronic devices that are applied to the skin need to be made from non-irritating biomaterials, and devices that are implanted must be manufactured to minimize the risk of irritating and damaging the surrounding tissue. Although irritating the nearby tissue is inevitable for implants, there are ways to decrease inflammation and prevent long-term initiation of the immune system. Another biocompatibility issue for implants is biofouling. This occurs when the implanted device activates the complement system, and macrophages and foreign body giant cells attach to the implanted device and grow, inevitably disrupting the normal operation of the device [33]. This response makes the devices cytotoxic and unstable, and renders bioelectronic devices, such as biosensors, unsuitable for long-term implantation [34].

Two ways to minimize the inflammatory response is to create ultrathin devices and/or devices with a low Young’s modulus to closely match the Young’s/elastic modulus of the tissue. Both increase the conformability of the device, allowing for accurate recordings [35]. Flexible and stretchable materials are less likely to inflame the nearby tissues and cause local swelling, which also lead to inaccurate measurements. Close contact with tissue also increases the surface area for the recording devices, which increases the accuracy of bioelectronics [5].

**Figure 1:**
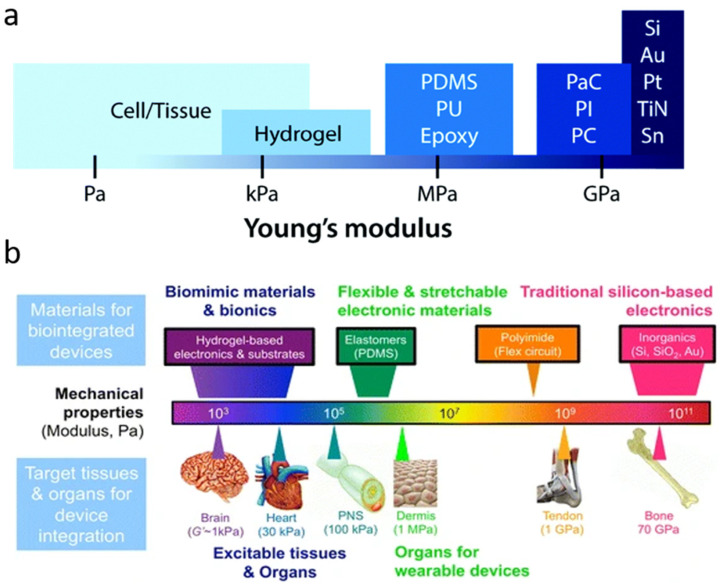
(**a**) Young’s modulus of multiple biomaterials compared to cells [37]. (**b**) Young’s modulus scale comparing multiple types of bioelectronics and the moduli of multiple tissue types in the human body [38].

Bioelectronics are usually made with flexible substrates and are encapsulated to protect the nearby tissue. To create compliant bioelectronics, the materials must have a low modulus. Human tissue has a modulus range between 10 GPa for bone to 1 kPa for some soft tissues. Other soft tissue, such as brain tissue, falls below 10 kPa [35]. Materials with a lower modulus allows for a better signal transduction [35]. Stretchable electronics must have high flexibility and should tolerate a tensile strain of at least 10% [36]. Plastics and polymeric materials show the most promise for creating clinically relevant flexible bioelectronics; however, there is still a mechanical mismatch between tissues and these flexible materials. To bridge that gap, research has shifted to using polymers with hard and soft segments and elastomers. For example, regular silicon (Si), a common substrate for bioelectronics, can have a modulus of about 130 GPa, as shown in Figure 1a. Silicone elastomers, such as PDMS (polydimethylsiloxane), have a lower Young’s modulus that is suitable for soft tissue (Figure 1b).

However, these materials are still not suitable for long-term implantation due to the mechanical mismatch between the non-stretchable polymers and the softer tissues. This mismatch causes inflammation that results in swelling, and thereby decreases the accuracy of the recording device. To combat this, research is aimed at creating hybrid materials with suitable chemical and mechanical properties by changing the chemical structure of these materials, i.e., adding hydroxyl groups to increase stretchability [39]. Mixing materials can result in a material with favorable characteristics from both materials. The most common example is adding an elastomer to a material to increase the stretchability [39,40]. Another method involves using a material with the wanted bulk properties and treating the surface of the material to include characteristics that immediately effect the surrounding environment. Surface treatments are performed to increase adhesion to other materials [41], to increase electrochemical properties, and to improve biocompatibility. One example of a surface treatment is the addition of a hydrophilic coating to a device to increase cell adhesion [42]. This section focuses on common materials and hybrids used to create the different components in flexible and stretchable bioelectronics, the substrate and encapsulation layers, and the conducting portions such as traces or recording electrode arrays.

### 3.1. Polymeric Substrates

Substrate and encapsulation materials are nonconducting materials. Substrates are used as the first layer for the electronics to be printed on and can be used to encapsulate internal components and or protect the entire device. These materials protect the components from mechanical deformation or environmental erosion caused by the body. In addition, the materials can provide adhesion for the device via van der Waals forces, covalent bonds, or hydrogen bonds. Polymers are commonly used as substrates because they are cheap to manufacture and are usually highly tunable.

**Figure 2:**
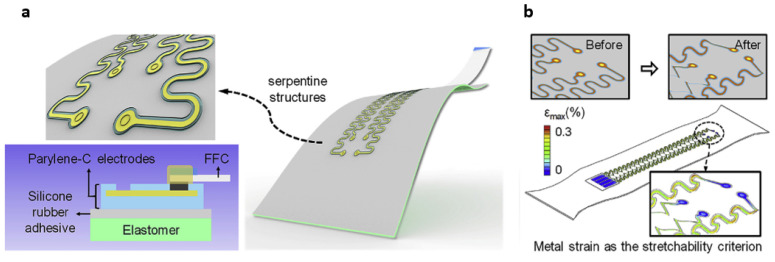
Stretchable Parylene-C electrodes. (**a**) A schematic of stretchable parylene-C electrodes with serpentine interconnects to increase the stretchability of the electrodes. A silicone rubber adhesive was used to stick the electrodes onto the elastomer substrate. Attaching the electrodes to an elastic substrate limited external deformation [43]. (**b**) A diagram of the strain applied to the electrodes when stretched [43]. Reprinted with permission from Ref. [43]. Copyright 2020 The Chinese Ceramic Society.

Thin-filmed parylene-C is a flexible polymer with an elastic modulus of about 3.2 GPa and a Poisson’s ratio of 0.4 [43]. Therefore, it is a good material for hydrophobic coatings around bioelectronics for in vivo and in vitro tests. Ji et al. used parylene-c to coat their electrocorticogram (ECoG) electrodes, which were constructed from nanostructured gold and conductive PEDOT [43]. A schematic of the design is shown in Figure 2. The conducting traces were made into a serpentine structure to increase its flexibility and stretchability, which is discussed in more detail in Section 3 of this review.

To support insertion, biodegradable silk fibrin was added to the stretchable parylene-C electrodes for easier surgery afterwards; the fibrin degraded over time. After six months of in vivo implantation in rat brains, 75% of these devices still showed a stable impedance for accurate recordings [44]. Currently, parylene-c is used as a substrate for neural electrodes due to its low modulus and chemical stability. However, in chronic implants, parylene-c can result in unfavorable biocompatibility. One study found that in in vitro samples aged one year, a tissue capsule around the device kept increasing in size for up to 16 weeks. This tissue capsule revealed macrophages and fibroblasts were in the area, but no cytokines were present after week 8 when initial inflammation was resolved [45]. The researchers of this study concluded that parylene-c induced more tissue deposition in the local area than the polymer polyimide and, for chronic implants, parylene-c was ruled out as a favorable substrate. However, their analysis also stated that the implant did not harm neural tissues nor was the efficiency of the neural device decreased after encapsulation. This encapsulation occurred because parylene-c is relatively more hydrophilic than polymide, which attracted more matrix deposition [45].

Silicones are polymeric siloxanes with a wide variety of use, from medical equipment and implants to houseware. Medical grade silicones and silicone rubbers are bioinert, water resistant, and biocompatible. Low-density silicone provides elasticity and flexibility with simple processing methods. Silicone can be used as a material for encapsulation. This material is most common for flexible and stretchable bioelectronics due to its diverse uses and easy fabrication method. It can also be bought commercially as Ecoflex, which has a low enough modulus for coating bioelectronic devices.

Another common silicone used in flexible bioelectronics is poly(dimethylsiloxane) (PDMS). PDMS is a biostable polymer that can be easily modified by embedding other substrates or manipulating its molecular structure [46]. PDMS is a favorable biomaterial because its surface and bulk modifications can result in several changes: changes in stiffness, the creation of anti-bacterial properties, and a short-term change into a hydrophilic polymer [47]. Conductive materials can be used inside of etched tracks to create flexible circuits. PDMS has a stretchability of up to 1000% [47]. Its flexibility is determined by its Si–O siloxane backbone. The average Young’s modulus is ~1–3 MPa. Super soft PDMS has a lower modulus of 0.1 kPa–2.3 MPa, which is favorable for soft tissues. Conductive materials such as silver, gold, carbon nanotubes, graphene, or aluminum oxide can be added into etched tracks within PDMS to create a functioning flexible and stretchable circuit. At the National Taiwan University, Chun-Yi Li and Ying-Chih Liao used plasma-treated PDMS to create a flexible, stretchable, and printable thin-filmed pattern. The film was plasma treated and a silica-like layer was added to the surface of the PDMS to increase the wettability. Finally, epoxy binder and silver paste were added to the silica layer as a printed pattern. These printed conductive films were bended, twisted, and stretched, all while retaining conductivity. Their fabrication method created a thin film with excellent mechanical stability [41].

**Figure 3:**
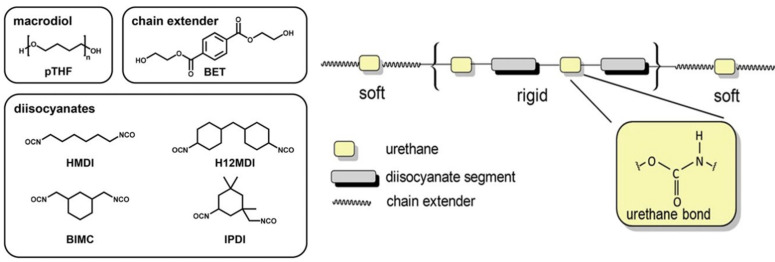
Chemical structure of polyurethanes. (**left**) The basic components of polyurethanes [49]. (**right**). Structure of the elastomer thermoplastic polyurethane which contains hard and soft segments [50]. Reprinted with permission from Ref. [50]. Copyright 2020 Springer Science Business Media.

The biomedical use of polyurethane (PU), specifically ether based [42], has gained more traction in this century and has become a common material for vascular grafts, due to its high tear and abrasion resistance. PU contains three components: a diisocyanate or triocyanate, a chain extender, and a macrodiol, as shown in Figure 3 [48].

PU is highly tunable when mixed with other polymers. Pure PU has a Young’s modulus of ~91 MPa and a stretchability of 290% [51]. As an elastomer, it has a high flex-fatigue life and a better mechanical degradation resistance compared to silicone elastomers [48]. The mechanical behavior of the elastomer is also tunable because its Young’s modulus is dependent on the temperature and humidity [48]. This further proves that PU is another organic material with multiple applications for flexible and stretchable bioelectronics. Elastomeric PU has even been advanced to be self-healing, with an elongation at break of 1900% [52]. Like PDMS, PU can be used as a base insulator to layer conductive tracts upon it. By adding silver flakes to pristine PU, Zhou Li and his colleagues were able to increase the conductivity of the flexible polymer for an adhesive [53]. One research group used polyurethane as a scaffold to hold 3D networks of the conductive material boron nitride. They used PU to increase the flexibility and stretchability of their thermally conductive composite film. This film did not hold electrical conductivity above strains of 40% after 100 stretching cycles, indicating breaks in the conductive paths [54]. The conductive materials chosen are an important part of any stretchable and flexible device. The type of material and its manufactured shape will determine how stretchable and reliable the device will be.

### 3.2. Conductive Materials

Conductive materials are imperative for bioelectronics to create conductive traces and the recording portion of the device. Metallic materials such as silver (Ag), copper (Cu), gold (Au), and carbon-based materials are highly conductive materials. However, these traces and electrodes must be flexible and stretchable to undergo repeated excessive strains without break. There are multiple avenues to create promising flexible and stretchable conductive materials: (1) hard metals can be manufactured into thin nanowires, (2) flexible materials can be infused in an elastic matrix, (3) conductive polymers or carbon-based materials can be used [55], and (4) by adding metallic flakes in a polymeric substrate, manufacturing ultrathin metallic plates, and liquifying metals [55]. Regardless of the method chosen, these materials must be able to remain electrically conductive and stable after withstanding several cyclic strains.

#### 3.2.1. Metal Nanowires

Creating nanowires is one way to make conductive materials stretchable and flexible for bioelectronics. Silver, copper, and gold are all promising materials for bioelectronics. Silver nanowires (AgNWs) have the highest electrical conductivity of all metal materials; however, due to the high cost of silver, they are usually avoided for creating bioelectronics. Gold nanowires (AuNWs) can also be used, but can also be costly. Copper nanowires (CuNWs) are most used due to the lower price and excellent electrical conductivity of copper. Conductive polymer nanowires (CPNWs) are also an excellent alternative to expensive metal NWs; one example is discussed later in this section.

CuNWs are highly conductive and low in cost but have poor dispersity. To combat this, Huang and his team infiltrated CuNWs into poly(styrene-block butadiene-block-styrene) (SBS), to create printable CuNW-based composites [55]. These printable composites were able to stretch and maintained conductivity after 1000 cycles of 4.0 mm bend radius. The printable nanowires had high electrical conductivity with a break elongation up to 920%. The elastic modulus of the wires was reported to be 0.081 GPa. The group only tested the composites on paper to create flexible circuits; however, this material is an excellent prospect for creating traces on stretchable and flexible substrates [56].

#### 3.2.2. Conductive Polymers and Conductive Liquids

Conductive polymers are excellent materials for flexible bioelectronics; however, they have limited stretchability because of their rigid backbones. One example is Poly(3,4-ethylenedioxythiophene): poly(styrenesulfonate) (PEDOT:PSS). PEDOT:PSS is a polymer with tunable electrical conductivity. PEDOT:PSS is the combination of the PEDOT monomer and the dopant, PSS. By applying a current over the mixture, the monomer oxidizes and the PSS becomes electrostatically bound to the polymer backbone [57]. Organic solvent doping treatments, ionic liquid treatments, strong acid soaking treatments, and acid-assisted transfer-printing are all treatments that can be used to increase the electrical conductivity of this polymer. PEDOT can be mixed with other polymers to create a flexible and stretchable conductive polymer. Hansen and his team created a conductive polymer mix of PSS and a PU elastomer polymer that increased in conductivity as it was being stretched, and decreased in conductivity when relaxed [58]. A blend of PEDOT:PSS and PDMS also proved to be a highly stretchable conductor [59]. Both these blends maintained electrical stability after strains; however, creating these composites proved to be challenging due to difficulty in phase separation [35]. Another way to increase the stretchability of PEDOT:PSS is to add multiple hydroxyl groups to the polymer during synthesis; researchers stated an increase in the elongation until break from <10% to >50% [39].

A number of problems remain to be addressed in the fabrication of printable circuitry before advancing. Some problems include the decrease in measurement accuracy after repeated stress and strain loads. This can be caused by the separation of the polymer and conductive materials, or microcracks creeping into the conductive tracts of electrodes. To combat the loss of conductivity in the electrodes, chemically stable liquid metals are favored for flexible and stretchable devices. Gallium-indium alloys are a common liquid metal to use for printable circuity. A self-healing, eutectic gallium-indium alloy (78 wt% Ga and 22 wt% In) was reported to have a low viscosity for fabricating the device and high conductivity [60]. However, using this alloy has a major problem during processing: when in the presence of oxygen, it is incompatible with most standard liquid processing techniques [60].

Polyaniline (PANI) is a conductive polymer popularized due to its low cost, tunability, environmental stability, high temperature resistance, and high electrical conductivity [61]. Its structure consists of benzene rings in the backbone, making the polymer initially non-stretchable. Pure PANI is highly soluble, and has poor mechanical properties, making it hard to create sensors with [62]. PANI’s electrical conductivity decreases over time and in physiological environments [63]. Despite this, PANI is an acceptable polymer for conductive traces. By electrospinning PANI into one-dimensional nanowires, researchers can increase its flexibility [63]. This polymer can also be woven into nanofibers to make the polymer stretchable. One application of this is a stretchable temperature sensor [64].

### 3.3. Carbon-Based Materials

Carbon-based materials are other conductive materials that can be used for traces or electrodes. Some carbon-based materials include graphene and carbon black. These materials are known for their highly conductive properties and their unique molecular structures.

#### 3.3.1. Graphene

**Figure 4:**
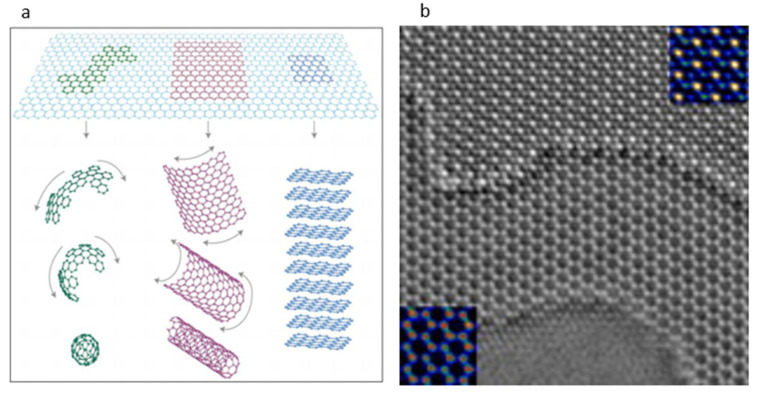
(**a**) Two-dimensional graphene sheets in various shapes: green shows 1D fullerene shapes, red shows graphene rolled into nanotubes, blue shows graphene stacked into 3D graphite [67] Reprinted with permission from Ref. [67]. Copyright 2007 Nature Publishing Group.; (**b**) atomic arrangement of single- and double-layered graphene [68]. Reprinted with permission from Ref. [68]. Copyright 2010 Elsevier Ltd.

Graphene is characterized as carbon molecules stacked in a monolayer honeycomb. Its Young’s modulus is 1 TPa. Graphene has excellent flexibility because the materials’ atoms are atomically thin; it also has some stretchability due to its hexagonal lattice structure [65]. However, the material’s conductivity can be destroyed by an elongation above 20%; thus, cross-linking graphene with an elastomer is important to enhance stretchability [66]. Graphene is commonly grown by chemical vapor deposition (CVD), which is discussed in the next section. Common metals used are nickel, iron, and copper. Different metals, temperatures, gas pressures, and compositions affect the number of layers and structure of the created graphene. For example, increasing solubility of carbon results in a thicker graphene film. Once formed, graphene is scraped off the metal and used to form different shapes: one-dimensional (fullerene), two-dimensional (nanotubes), and three-dimensional (graphite) (Figure 4a) [65]. Carbon nanotubes and graphene flakes can both serve as conductive fillers or base conductive material. Graphene can be woven into textile materials to increase conductivity [47].

Graphene is a carcinogen and can be toxic to the liver, kidney, brain, and a few other organs. It can also initiate chronic inflammation resulting in granulomas, or local buildup of immune cells, once accumulated in the body [69]. Cytotoxicity has been found to be related directly to the morphology and concentration of graphene [69]. Therefore, when used in bioelectronics, graphene must be encapsulated or avoided. Another reason graphene is not often used is its high cost. Further research and testing of different modifications are key to its increased use as a biomaterial [69].

#### 3.3.2. Carbon Black

Carbon black (CB) is a carbon-based material [70] that is preferred to graphene due to its low cost and conductivity. It is found in soot and is biocompatible due to its spherical shape [71]. CB is amorphous or shapeless carbon. Unlike graphene, it does not have a defined crystal structure. Since CB does not have a structure, it can be mixed with other polymers to make it stretchable and flexible. CB and Ecoflex can be mixed to create electrodes. These electrodes have been printed on a barium titanate elastomer composite, and maintained stability through 1000 cycles of 100% strain [71]. Another application used CB nanoparticles mixed with Ecoflex to create conductive traces inside more Ecoflex to create a stretchable nanocomposite piezo-resistor, decreasing resistance with increased strain [72].

Although all the biomaterials mentioned in Table 2 have been safely tested with regards to the human body, surface modifications or encapsulation are still important to increase device longevity. These favorable device characteristics mentioned are for both implantable and wearable electronics. Wearable devices should have high mechanical strength to withstand the outside wear and tear, and must be biocompatible to prevent skin irritation. For implantable devices, however, the body’s environment can be detrimental to any foreign object. Body temperature, pH, and blood components can all increase the breakdown and release of ions from the implanted materials.

## 4. Engineering Organic/Inorganic Functional Materials in Stretchable Bioelectronic Devices

Flexible/stretchable devices for wearable and bio-integrated electronics not only require a flexible/stretchable substrate, but also electronic systems that possess mechanical flexibility, stretchability, and conformal features, in addition to excellent electrical properties. High-throughput, large-scale, and cost-efficient fabrication of flexible and stretchable bioelectronic devices requires a novel approach to material design, sensing material, fabrication technique, and sensing mechanism [84]. To achieve stretchable bioelectronic interfaces, several attempts to merge the properties of high stretchability of elastomer and high conductivity of organic/inorganic conductors have been made. Flexibility and stretchability in conductive materials can generally be achieved by using (1) geometrically soft conductors through the use of different structural configurations, (2) conductive polymer composites, or (3) conductive liquids [85].

The conductive materials listed in Section 2 can be either hard materials or intrinsically stretchable materials. As mentioned earlier, hard materials such as pure metals (Ag, Cu, Au, etc.) offer the advantage of having high conductivity for efficient signal transduction and high sensitivity. However, those inorganic materials are rigid and do not possess mechanical flexibility and stretchability. Processing these materials into thin films and nanowires (NWs) using methods such as chemical vapor deposition (CVD) and lithography add the property of flexibility, but not stretchability [86,87]. Hence, researchers developed a strategy where they couple mechanically guided structural designs on those materials to accommodate large deformation, and embed those patterned materials in elastic substrate. Common mechanically guided structural configurations, i.e., serpentine geometry, wavy/wrinkled structures, and fractal motifs, are discussed in Section 3.1.

**Figure 5:**
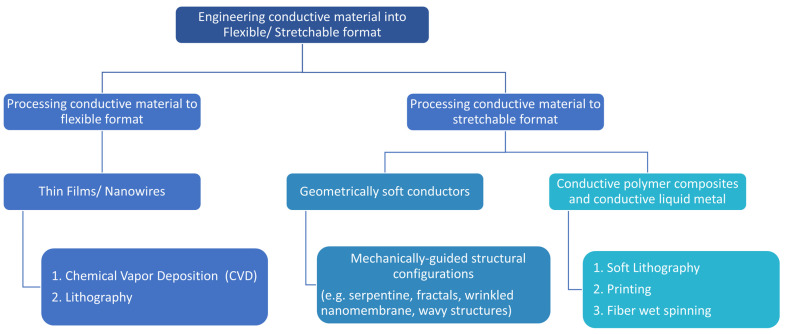
A chart summarizing the engineering of conductive material into flexible and stretchable format.

Polymer composites with conductive fillers (e.g., carbon black, graphene flakes, metal NWs, carbon nanotubes, metal nanoparticles), and liquid metals, when processed into thin films, can absorb mechanical deformation by themselves and are considered to be intrinsically stretchable materials [88,89]. To process those conductive polymer composites and liquid materials into thin films, several fabrication techniques, such as soft lithography, printing, and wet spinning, are utilized, and are discussed later in the section. In addition, coating, dipping, and transfer printing can also be utilized to achieve thin films from conductive liquids. A chart summarizing the processing of these conductive materials into a flexible/stretchable format is shown in Figure 5.

### 4.1. Structural Configurations to Render Stretchability to Hard Inorganic Materials

**Figure 6:**
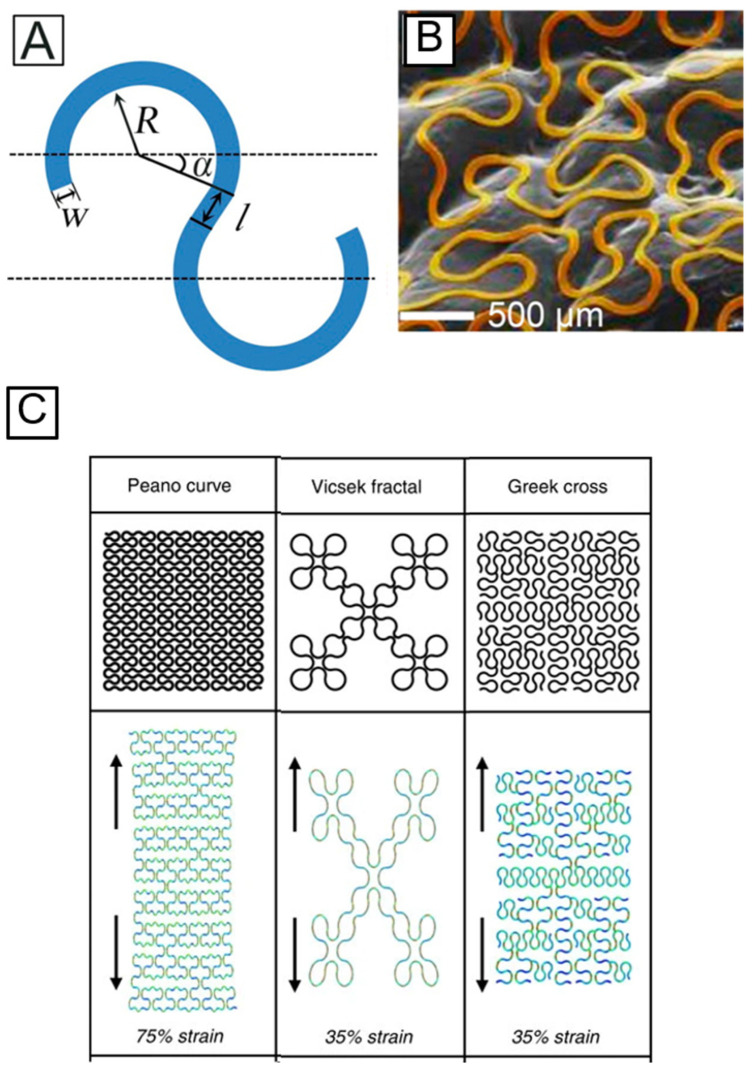
Representative geometrical configurations for hard–soft material integration. (**A**). Schematic representation of a serpentine unit cell. (**B**). Example of serpentine structured traces on an electrode array [91]. Reprinted with permission from Ref. [91]. Copyright 2015. American Chemical Society. (**C**). Different patterns of fractal-inspired layouts of metal wires (top), and FEM images of each structure under elastic tensile strain [90]. Reprinted with permission from Ref. [90]. Copyright 2014 Nature Publishing Group.

To transform the non-stretchable electrically conductive material into a stretchable format, researchers use the strategies of mechanically-guided structural configurations. Creation of different structural configurations, such as serpentine geometry, wrinkled nanomembranes, wavy structures for geometrically soft conductors, and fractal layouts, have aided in dramatically improving the stretchability of devices. Conductive traces can be deposited into different shapes, such as curvilinear shapes with arc sections. Integrating arc sections instead of sharp turns into an electrical wire improves the elastic mechanics [90]. One example of these curvilinear shapes is the serpentine structure. Serpentine geometry (Figure 6B) enables the in-plane stretchability by minimizing the maximum tensile strain (ε_max_), which is strongly dependent on the adhesion with a stretchable substrate [91]. In a serpentine structure, four geometric parameters—width (w), radius (R), arc angle (α), and arm length (l) (Figure 6A)—dictate the stretchability. Lee et al. concluded that stretchability increases with a small w/R ratio, large l/R ratio, and large α [91]. Additionally, researchers have created stretchable electronics by placing the semiconductor material on a pre-strained elastomeric substrate. Such devices undergo a lateral compression upon removing the pre-strain, and therefore attain out-of-plane wavy or wrinkled structures. This approach has been exploited to fabricate stretchable organic photovoltaics [91]. Fan et al. demonstrated a promising approach to integrate electronic material in soft elastomeric substrates [90]. The method involves patterning the metal wires in various fractal motifs. The topology types selected for creating those fractal motifs were lines (Peano), loops (Vicsek), and branch-like meshes (Greek cross) (Figure 6C). These fractal constructs consist of a high-order iteration of lines, loops, or meshes that are linked together. Those fractal constructs, unlike the serpentine geometry, allow the researchers to control and tailor the stretchability of the electronic devices depending on their applications. The orientation of the pattern enhances the device’s elastic strain in the selected direction, thereby providing the ability to accommodate different types of deformation modes from uniaxial and biaxial, to radial deformation modes. The enhancement of stretchability can be attributed to factors such as the geometric scaling of the arc sections, increase in the length of the wires, and addition of high-order spring-like motifs. These fractal-based layouts have the potential to be used for radio frequency-based applications, and offer a promising approach to devise MRI-compatible skin-mountable or implantable devices.

### 4.2. Chemical Vapor Deposition for Creating Thin Films

**Figure 7:**
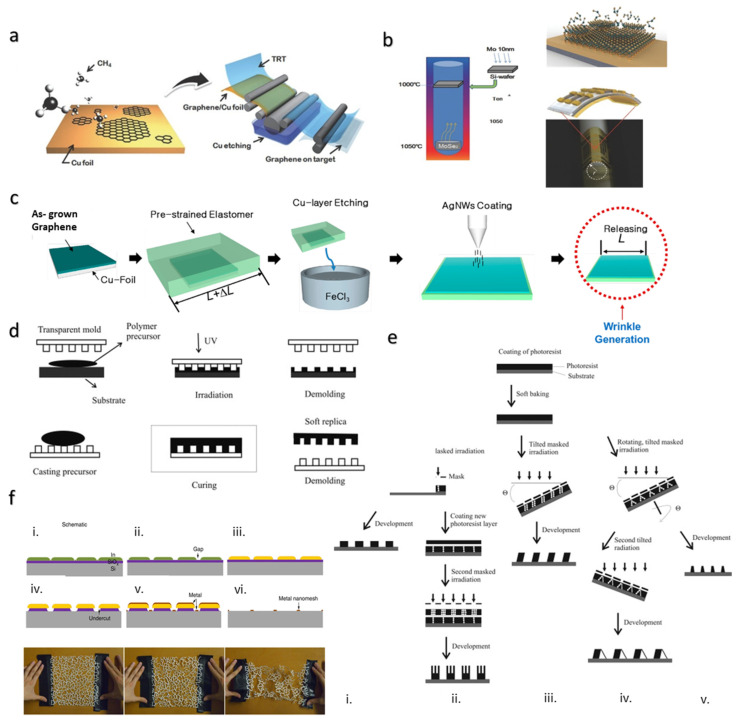
Fabrication methods for stretchable electronics. (**a**–**c**) Chemical vapor deposition (CVD) (**a**). Schematics of the synthesis mechanism of CVD graphene on Cu foil (left) and the roll-to-roll transfer process of graphene (right) [95]. Reprinted with permission from Ref. [95]. Copyright 2013 American Chemical Society. (**b**). Modified CVD (mCVD). Schematic representation for synthesizing multilayer MoSe_2_ film. Schematic cross-section of a collection of MoSe_2_ transistors on a flexible PI/PET substrate [94]. Reprinted with permission from Ref. [94]. Copyright 2016 Wiley-VCH Verlag GmbH & Co. (**c**). Fabrication process of the wrinkled graphene-AgNW hybrid electrode [96]. (**d**–**f**) Lithography method. (**d**). UV Nanolithography (top) and soft lithography (bottom) [97] (**e**). Photolithographic methods using masked irradiation and a negative photoresist material: (i) Patterning by single exposure, (ii) patterning by layer-by-layer coating and exposure, (iii) tilted patterning by single inclined exposure, (iv) patterning by double inclined exposure, (v) tapered patterns by rotating tilted exposure [97]. Reprinted with permission from Ref. [97]. Copyright 2011 Elsevier Ltd. (**f**). Fabrication of highly stretchable and transparent nanomesh electrodes via grain boundary lithography [98]. A sheet of paper is laser-cut using the magnified image of Au nanomesh. The left image shows without any cutting. The middle image shows the stretching of the structure after cutting a few ligaments. The right image shows the stretching of the structure after cutting more ligaments. Reprinted with permission from Ref. [98]. Copyright 2014 Nature Publishing Group.

Chemical vapor deposition (CVD) is a widely used manufacturing process in the semiconductor industry to deposit or grow thin solid film on the substrate held in a reaction chamber. This process has been extensively used to grow large-area and high-quality graphene film on metal [92]. During CVD, the metal substrate to be coated is exposed to a gaseous carbon source, such as methane, acetylene, or methanol. The decomposed carbon radicals from these gases precipitate out on the metal substrate to form layered graphene. Commonly used metal substrates are Ni and Cu, which also act as a catalyst for the reaction. Properties such as the number of layers, size, morphology, orientation, doping, defect, and grain boundaries can be controlled by manipulating parameters such as the temperature, chamber pressure, gas flow rate, and source–substrate distance [93]. High-quality and sensitive 2D materials require few grain boundaries or large single crystals to reduce charge scattering at the grain boundary and enhance the electronic, mechanical, and thermal properties of the 2D material [93]. This challenge of growing large-grain crystals was addressed by Rhyee and team using modified chemical vapor deposition (m-CVD) for the fabrication of high-mobility 2D-layered transistors based on large-grain and highly crystalline Molybdenum Diselenide (MoSe_2_) films grown onto SiO_2_ substrates (Figure 7b) [94]. The mechanical flexibility of these thin film transistors was realized by transferring the MoSe_2_ onto the flexible polyimide (PI) substrate. The fabricated transistors have applications in human robotics and human-centered soft electronics. Other challenges in growing high-quality large film include the random layer distribution, variation in growth at different areas, transfer of the 2D film produced from the growth substrate to the target device substrate, and unavoidable damage and contamination during transfer of the film to the target [93].

CVD aids in growing thin film on substrate. Flexibility and stretchability can be realized by transferring the thin film onto flexible/stretchable substrate. To realize the stretchability on the device, it is ideal if the graphene layer can be grown directly on the stretchable polymer substrate. However, one of the challenges of using the polymer substrate for direct growth of graphene is that it possesses low processing temperature, high surface roughness, and a low thermal expansion coefficient; therefore, it is not an appropriate substrate for conventional CVD. Unlike CVD, plasma-enhanced CVD (PECVD) possesses a low processing temperature, thereby providing an opportunity for direct growth of graphene on flexible/stretchable substrate. However, its disadvantage is the production of inferior graphene. Due to the inferior graphene qualities resulting from PECVD, Jang et al. reported coupling CVD for growing graphene film on Cu with the roll-to-roll transfer method to polymer substrate (Figure 7a) [92]. Similarly, atmospheric pressure chemical vapor deposition (APCVD) coupled with the transfer method and inkjet printing was also used for the fabrication of a conductible vertically aligned carbon nanotubes (VACNTs)/PDMS/Graphene Oxide (GO) stretchable electrode [99]. It was observed that after multi-cycle stretching the electrode up to 100% tensile strain, it did not show an increase in sheet resistance [99]. Fabricating a highly stretchable wrinkled graphene for a sensitive strain sensor and a smart window was also reported using the CVD approach for growing graphene, and the transfer printing method for transferring the graphene onto a pre-stretched elastomer (Figure 7c) [96,100]. Chen et al. incorporated crumples and buckles on graphene by attaching the graphene film to a uniaxially pre-stretched very-high-bond (VHB) film to support the stretchability [100]. The gauge factor, defined as the ratio of ΔR/R_0_ to ε (resistance variation to applied strain), was 20.1 at the working range of 105% tensile strain. Additionally, with small strains (ε) < 105%, the resistance increased slowly due to the buckled graphene structure. However, increasing the strain >105% led to formation of cracks, which resulted in a rapid increase in resistance. In summary, CVD coupled with the transfer method is generally utilized to engineer flexible/stretchable devices with high graphene quality.

### 4.3. Lithography Method for Creating Thin Films and Nanowires

Lithography is a popular key technology in the semiconductor industry used for the fabrication of integrated circuits and microchips. This approach is utilized to fabricate a desired pattern ranging from nanometer to micrometer size on the underlying substrate. This fabrication approach has been extended to fabricate soft and stretchable electronics for biomedical applications, such as skin-mountable and wearable electronics. Photolithography, soft lithography, nanoimprint lithography, e-beam lithography, focused ion beam lithography, scanning probe lithography, etc., are some of the widely used lithography techniques. Commonly used photolithography is a masked UV lithography that uses a positive or negative photoresist (Figure 7d). Upon exposing the negative photoresist to UV light using a photomask, the UV-light-exposed areas become polymerized, and therefore become insoluble in developer solution. On the contrary, upon UV exposure using a mask, the positive photoresist areas become depolymerized and are therefore easily dissolved using developer solution. Generally, the photolithography process comprises of several steps, such as photoresist application, prebake, mask alignment and exposure, development, post bake, etching, and resist stripping (Figure 7e) [97]. Electron beam lithography, in contrast, is mainly used to produce a photomask. It incorporates a high-resolution direct writing approach using an electron beam on an electron-sensitive resist, and is therefore not a suitable approach for mass production [97]. The soft lithography process enables fabrication of structures or the transfer of material using an elastomeric stamp, mold, or photomask (Figure 7d). This process is easy, fast, low-cost, mass reproducible, and can be used for thin film fabrication.

Although lithography enables the deposition of metal on flexible/stretchable soft substrates, the problem of crack formation on conductive metal conduits upon stretching or bending the device remains. In addition to this technique, researchers use the strategy of structural configurations to realize a highly stretchable device while keeping the conductive materials intact to maintain the device’s conductivity. When engineered with serpentine-shaped ligaments, metal films tend to become highly stretchable, as mentioned in Section 3.1. This strategy of enhancing stretchability was presented by Guo et al. by fabricating Au metal nanomesh electrodes using a method called grain boundary lithography [98]. This lithography method involves depositing Au metal in undercuts created in the SiO_x_ and In_2_O_3_ layer by isotropic etching followed by the lift-off process (Figure 7f). Au nanomesh on PDMS substrate resulted in only a slight increase in resistance when it was stretched to about 160% strain, or after 1000 cycles at a strain of 50%. This high stretchability due to the serpentine-shaped ligaments is attributed to the distributed rupture of the Au ligaments and the out-of-plane deflection of the serpentine ligaments. The nanowire approach of fabricating stretchable sensors has also been exemplified by the research conducted by Tiefenauer. Tiefenauer’s team utilized UV interference lithography for fabrication of a nanowire array template on a PEN/PVA sheet, followed by gold evaporation onto those templates and template stripping-based nano-transfer printing of stretchable plasmonically coupled graphene structures on an elastomer for Surface Enhanced Raman Scattering (SERS)-based sensing applications [101]. Another approach for highly stretchable low-impedance electrode fabrication comprises microcracks, as demonstrated by Decataldo et al. The team reported microcracked Ti/Au films covered with stretchable conducting polymer composite fabricated via the method of lithography and electrodeposition for bioelectronic recording from small peripheral nerves. The composite displayed high conductivity under tensile strain exceeding 40% [85].

This section has discussed the deposition of hard metal onto a substrate using lithography, and the realization of stretchable devices using either structural configurations or transfer printing on a stretchable substrate. An alternative approach to realize soft and stretchable microelectronics using lithography is the use of liquid metals such as eutectic gallium-indium (eGaIn) alloy. EGaIn alloy is nontoxic, possesses excellent mechanical, thermal, and electrical properties, and facilitates a broad range of patterning methods, including soft lithography based on stamping, stencil printing, injection, and additive and subtractive patterning processes. Lithography-enabled stencils have a low resolution (≈20–200 µm) and pose challenges such as having a rough surface and loss of material during lift-off. The microfluidic injection technique provides a higher resolution (>10 µm) but requires microchannel thickness >50 µm. The subtractive technique is a low-cost and facile method, but the material removal process is slow in the case of patterning small features on large substrates. Additive patterning, like direct writing, has a low resolution (≈100 µm) due to the size limitation of the injection nozzle. On the contrary, feature sizes >2 µm can be achieved via soft lithography [102]. Therefore, a good selection of lithography method is required to achieve high-quality stretchable devices using liquid metals. A potential solution to overcome patterning challenges is employing wetting/nonwetting physical or chemical surface modifications, which help by providing smooth and uniform deposition of thin films for multiscale patterning and functional integration [102]. By combining additive and subtractive soft lithography with surface modification and 3D heterogenous integration, Kim and team successfully fabricated eGaIn thin film-based functional soft microsystems such, as a soft LC (inductor-capacitor) sensing platform, a fingertip-mountable biological sensing platform, and soft heaters with heating capability. The fabricated soft and stretchable circuit exhibited bending and strain deformation up to 50% while maintaining electrical conductivity.

### 4.4. Printing for Creating Thin Films of Conductive Liquids

Chemical vapor deposition (CVD) aids in fabrication of high-quality thin films of graphene with low flexural rigidity on a metallic substrate. PECVD, by comparison, can be used for direct fabrication of thin films of graphene on polymer for engineering stretchable devices; however, it cannot produce high-quality graphene. Hence, in order to engineer conductive materials to a stretchable format, researchers have to perform a film transfer from the growth substrate to a target stretchable substrate. Therefore, this does not represent an ideal fabrication procedure for the fabrication of a stretchable device. Although lithography can be used to create a conductive circuit pattern having a size in the range of nm to µm on a soft stretchable substrate, it requires a planar substrate surface, a photomask (for mask lithography), and expensive resources, such as a mask aligner, to develop complicated structures. Moreover, due to phenomena such as diffraction and the wavelength factor of optical sources, the lithography method may not produce high resolution patterns on the substrate, and may also limit the method. Similarly, serpentine and wavy structures of rigid materials limit the strain of devices and can lead to the formation of cracks and fractures. Hence, conductive liquid metal is an emerging and attractive field of research.

Printing is an additive manufacturing process that is a fast, accurate, and fully automated non-contact approach that uses liquid metal to create a conductive functional region on a single platform [60]. In addition to the robustness, flexibility, and stretchability of the device, printing also improves the repeatability and scalability of liquid metal processing, and provides the capability of achieving electronics with complex patterns without compromising the electrical conductivity of the devices [60].

To fabricate conductive electronics using printing techniques, it is pivotal to formulate conductive liquid metal ink with ideal features, such as the choice of the conductive material and the solvent, and the appropriate viscosity, surface tension, and rheology for achieving high printing performance and high accuracy [103]. A range of ink formulations have manifested the potential to be employed for printing. Some of those formulations are the commercially available AgNP ink [104,105], eGaIn liquid metal ink [60,106], toluene-based ink composed of Semiconducting Single Walled Carbon Nanotubes (SC-SWCNTs) and conjugated polymer (to prevent CNT aggregation) [107], poly (vinylidene fluoride-co-hexafluoropropylene) (PVDF-HFP) in N-methyl-2-pyrrolidone anhydrous [107], and poly(3,4-ethylenedioxythiophene):poly(styrenesulfonate) (PEDOT:PSS) in solution of bis (trifluoromethane) sulfonimide lithium [107]. These inks were prepared either by dispersion [104] or sonication [60,107]. Depending on its viscosity and rheology, the liquid ink may tend to emerge as a ball rather than a stream. The rheology of printable ink can be regulated for the jetting stream using additive agents or by optimizing the concentration of the ink [103]. Surface energy mismatch between the substrate and the ink results in poor wettability, which can be improved by decreasing the surface tension. The effective ways to regulate surface tension are either by performing oxygen plasma or UV/ozone surface treatment, by adding surfactants such as Triton X-100 or Zonyl FC-300 [41,103], or using toluene [101]. Printing of conductive liquid is mostly followed by the sintering process to create a conductive path. Sintering can be undertaken in several ways—self-sintering [108], mechanical [60], thermal [109], chemical [110], or using pulsed light [111] or a focused laser beam [105,112]. In addition to the robustness, flexibility, and stretchability of the device, printing also improves the repeatability and scalability of liquid metal processing and provides the capability of achieving electronics with complex patterns without compromising the electrical conductivity of the devices [60].

**Figure 8:**
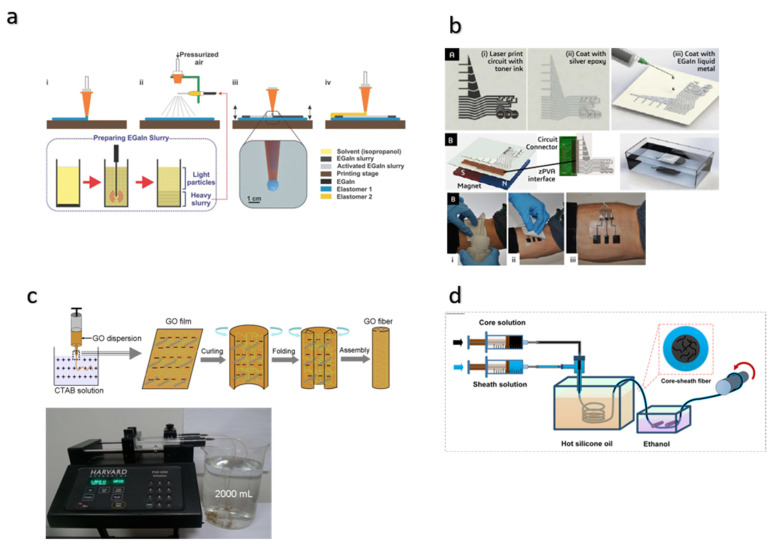
Alternative fabrication methods. (**a**,**b**) Printing method. (**a**). Schematics of printing of liquid metal (eGaIn) on an elastomer base layer [60]. (i) Extrusion printing of a base elastomer layer (ii) Spray printing of liquid metal (iii) Selective activation of the electrical path (iv) Extrusion printing of an encapsulation elastomer to seal the device. Reprinted with permission from Ref. [60]. Copyright 2017 Wiley-VCH Verlag GmbH & Co. (**b**). Fabrication steps for hydroprinted electronics. Process for application of a hydroprinted electronic tattoo over the forearm for EMG signal acquisition [113]. Reprinted with permission from Ref. [113]. Copyright 2018 American Chemical Society. (**c**,**d**) The wet spinning method of fabrication. (**c**). Schematic illustration of wet spinning GO fibers. Wet spinning set-up [118]. (**d**). Schematic illustration of the coaxial spinning process for highly stretchable fibers [122]. Reprinted with permission from Ref. [122] Copyright 2018 American Chemical Society.

Based on this knowledge about printing techniques, their advantages, and the considerations to be made when formulating conductive inks, next we look at how researchers have utilized this technique to fabricate stretchable devices. Mohammed G. Mohammed combined inkjet printing for the extrusion printing of uncured base elastomer, i.e., Smooth-Sil 950 layer and spray printing of eGaIn (78% Ga, 22% In) liquid metal ink prepared by sonicating bulk liquid metal in a carrier solvent (Figure 8a) [60]. Selective mechanical sintering of slurry particles was performed to create a conductive path. Lastly, extrusion-printed Sylgard 184 was used as an encapsulating elastomer. Lopes reported a novel method for rapid fabrication of a thin-film circuit with integrated microelectronics using a printing method and hydrographic transfer [113]. Highly conductive circuits were produced using silver paste with eGaIn coating on a non-conductive circuit template printed on Transfer Tattoo Paper (TTP) using a desktop printer (Figure 8b). Hydrographic transfer, also referred as water transfer printing, was demonstrated to fabricate a stretchable circuit for its application in human–machine interface devices (Figure 8b). Albrecht’s team fabricated highly conductive and highly stretchable (up to 300% strain) wires on plasma-treated PDMS using simple inkjet printing and commercial AgNP ink [104]. Lopez et al. fabricated stretchable Field Effect Transistor (FET) arrays and interconnections that can bear in-plane stretching of up to 20% strain by inkjet printing using PEDOT:PSS, PVDF-HFP, and SC-SWCNTs [107].

### 4.5. Wet Spinning Method for Flexible/Stretchable and Conductive Fiber Production

Due to a huge interest in flexible/stretchable electronics, the stretchable property of everyday textiles has been exploited as a platform for wearable electronics. Fiber is the fundamental unit of the textile. With the incorporation of conductive material, the fiber’s functionality can be expanded to stretchable strain sensors, supercapacitors [114,115], nanogenerators [116,117], etc., for wearable electronics. These fiber-like electronics not only provide excellent wearable properties, but also can withstand mechanical deformations such as bending, folding, twisting, and stretching. Such wearable electronics can be easily attached conformably onto rough and uneven surfaces such as human skin. As a result of the benefits of fiber-like electronics, researchers developed several strategies, one of which is the wet spinning method. The fiber wet spinning method is a facile and viable strategy to fluidly spin the macroscopic fiber in a continuous way [118]. The overall process of preparing conductive fibers includes preparation of dispersion, preparation of the coagulation bath, and wet spinning. First, the dispersion solution is loaded into a syringe pump or a spinneret. The dispersion is then extruded through the spinneret into a non-solvent bath for coagulation. The fibers are collected in the roller and dried at room temperature to remove the residual solvent in the fiber [119,120]. Post-processing is generally carried out to enhance the conductivity, including modification of the formulation with an organic solvent or conductive additives [121]. However, studies have been conducted to develop a fast one-step process and eliminate the post-treatment [121]. Zhang et al. demonstrated the production of strong and conductive PEDOT:PSS fiber in a one-step process by replacing the conventional coagulation bath with concentrated sulfuric acid [121]. Cong et al. reported the processing of graphene oxide (GO) dispersion in a coagulation bath of hexadecyctrimethyl ammonium bromide (CTAB) solution followed by chemical reduction to produce graphene fibers with mechanical strength of 182 MPa and conductivity of 35 S/cm (Figure 8c) [118]. The team also showed that soaking GO fibers in epoxy resin solution can produce fibers with good rigidity, and integrating poly(N-isopropylacrylamide) (PNIPAM) in GO dispersion can be undertaken to retain the thermosensitive property of PNIPAM in the fibers. The assembly mechanism of GO fibers is attributed to the electrostatic interaction between the CTAB and GO sheets. Tang et al. expanded this fabrication strategy to develop one-step coaxial wet-spinning assembly to fabricate silicone-elastomer-wrapped multiwalled CNT (MWCNT)-based core-sheath fiber with high stretchability above 300% and excellent stability (>1000 cycles) (Figure 8d) [122]. Seyedin et al. successfully demonstrated the wet spinning technique using polyurethane (PU) elastomer, PEDOT:PSS-conducting filler, dimethyl sulfoxide (DMSO) solvent for stable dispersion, and 80/20 *v*/*v* isopropanol/water mixture as a coagulation bath [123]

Some of the considerations required during fabrication are that the spinning ink must exhibit shear-thinning behavior for efficient flow, and an appropriate coagulation bath should be chosen so that the extruded spinning ink is rapidly vulcanized without any breakup [122]. The diameter of the fibers must be easily controlled by the nozzle size of the spinneret [119]. The fibers intended to be used as strain sensors need to have a high sensitivity for sensing tiny deformations during respiration, heartbeat, and speech recognition, and a broad sensing range to detect large movements, such as finger bending, walking, and running. In order to achieve both features, dip-coating of fibers has been reported [124,125]. A summary of advantages and disadvantages of each of the fabrication methods to create a flexible/stretchable device is presented in Table 3.

## 5. Transducers and Communication

As technology advances, the demand for new and innovative forms of wireless communication increases. These new forms of communication are being incorporated within polymers to help aid the medical field with progressive monitoring and tasks. They operate under different dynamic ranges, standards, and levels of repeatability, noise, and hysteresis, depending on the location of the device in use. Throughout this section, various methods of data collection and transition are reviewed in terms of the different types of transducers and how they enable communication. Transducers enable the conversion of one form of energy into another—usually electrical—to allows the devices to communicate. Various types of transducers also have different dynamic ranges [131]; the higher the range, the more precise and sensitive the transducer. The use of sensors for communication has aided the fields of biomechanics, clinical medicine, ergonomics, and biomedicine. 

When dealing with wireless forms of communication, electrical conductivity is essential for high gain with efficient bandwidth. The material used also needs to have resistance to various forms of degradation from the mechanical stress of use. The proper base material is also important regarding the use of the device. Does the situation call for a flexible or stretchable substrate? Although there are various forms of transducers and communication methods, the following sections discuss the prominent forms used, in addition to examples being used currently in the field of flexible and stretchable bioelectronics.

### 5.1. Piezoelectric Transducers

Since being first reported in 1880 [132], piezoelectricity has been used successfully in the design of therapeutic systems and widely used due to its transducer properties. To obtain an electrical charge, a force is first exerted on a given solid material. The stress upon the given material causes a deformation in the positive and negative subatomic particles of the material, causing a polarization that generates an electric field that is used to transform mechanical energy into electrical energy [133]; that is, a molecule is unperturbed with no polarization until an outside force exhibits stress upon the molecule. When stress is applied, it causes movement within the electrons and results in polarization. When the force is removed from the material, the polarization also stops. Due to its small size and high-frequency response, and versatile flexibility, the piezoelectric transducer is easy to use and excellent for clinical applications. This has resulted in therapeutic applications in the fields of neurosurgery and cancer treatment. In 1949, Dr. George Ludwig first reported the use of ultrasound in animal tissue [132] due to the piezoelectricity effect.

**Figure 9:**
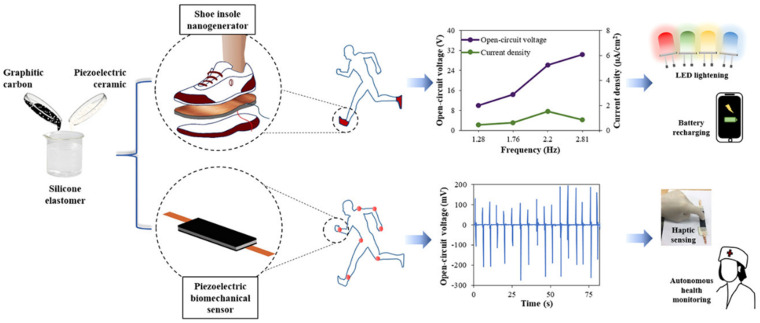
The proposed shoe-insole nanogenerator that can generate an open circuit voltage of ~27 V. The sensors used for movement detection responded to almost every joint movement [136]. Reprinted with permission from Ref. [136]. Copyright 2020 American Chemical Society.

As a result of new and emerging biomechanical technologies, piezoelectric sensors are used to measure either kinetics or kinematics. Applications for the use of piezoelectric transducers include their use in recording the forces between different parts of the body and support surfaces [134], uses in clinical settings, helping to monitor human performance, or even their use for leisure purposes. In the 1980s, due to their low cost and lightweight properties, piezoelectric transducers were used in the measurement of vibration and impact in activities. Pedobarography has grown significantly, in part because of the assistance of accelerometers and gyroscopes, which have enabled ambulatory measurements, thus leading to new advances in the study of gait and pressure while moving [134]. Some of the first uses of piezoelectrical materials in this fashion were first described by Hennacy and Gunther in 1975 in the measurement of dynamic pressure distribution [135]. Iqra Choudhry et al. described a flexible piezoelectric transducer that is capable of harvesting energy from human kinematics using flexible nanocomposite piezoelectric nanogenerators [136] (Figure 9). The group designed a shoe-insole nanogenerator with the aim of detecting human movement. In the design, movement generated power which, in turn, created a frequency that was transmitted to a digital storage oscilloscope and recorded through data acquisition (DAQ).

A recent monitoring device was developed specifically using the piezoelectric effect. This device, developed by Dagdeviren et al., is used for monitoring the vital signs and ingestion within the GI tract [137]. The device offers the flexibility and durability needed in the continually mocking GI tract. This unique device offers an interesting means of implantation—the user swallows an encapsulated device. Once inside the stomach, the capsule dissolves, releasing the monitoring device, which then unfolds and positions itself within the mucosa. Due to the piezoelectric nature of the device, as the mucosa causes the device to move, electricity is developed. The device uses hook-up wires to transmit the recorded movements via signals to a computer and a USB multimeter.

Based on the understanding of the need for stretchable devices, researchers have utilized either intrinsic piezoelectric materials (polyvinylidene fluoride (PVDF), polyvinylidene fluoride-trifluoroethylene (PVDF-TrFE) and odd nylon), piezoelectric electret, or piezoelectric fillers (barium titanate (BTO), lead zirconate titanate (PZT), (1−x)Pb(Mg, Nb)O_3−*x*_PbTiO_3_ (PMN-PT)) in a stretchable substrate such as PDMS or Ecoflex [138]. Piezoelectric electric materials exhibit piezoelectric effects due to the polarity generated from charged air bubbles or space charges injected by a corona charger at a high DC voltage [139]. Because these piezoelectric materials are flexible when processed into thin films, but not stretchable, researchers employ the method of structure configurations to provide the flexible materials with stretchability. Sun et al. reported utilizing the kirigami approach to fabricate stretchable strain sensors from flexible PVDF film encapsulated in PDMS [140]. The strain sensor could be used on curved surfaces, including the heart and body joints. Similarly, a highly stretchable PVDF vibration-sensing e-tattoo for siesmocardiography (SCG) monitoring was created utilizing serpentine interconnects on Tegaderm substrate [141]. Enrico and team created a press sensor made of composite material with PDMS and PZT fillers [142].

### 5.2. Ultrasonic Transducers

By interpreting reflected signals, similar to that of radar or sonar, ultrasonic transducers offer a low-power form of communication. Based on the properties of piezoelectric transducers, ultrasonic transducers can convert alternating current into ultrasound, in addition to reversing it. To produce an ultrasonic sound, piezoelectric crystals have an electrical current applied to them so their shape changes; this causes a vibration that creates sound waves in the same manner in which bats use echolocation. With the advent of microelectromechanical systems, new capacitive micromachined ultrasonic transducers (CMUTs) have emerged, helping replace some of the bulker piezoelectric transducers that some traditional medical machines have relied on [143]. CMUTs offer little impedance of the membrane, which vibrates to detect ultrasonic waves, and provide a wide bandwidth and an excellent coupling efficiency. A unique use for CMUTs was developed by Ramanaviciene et al., who created a biosensor that is able to sense the change in mass loading on the top of a membrane, which was then used to detect bovine leukemia virus proteins [144]. The device read the normal frequency of the CMUT and, with any deviation caused by the change in device, the results were then recorded and transmitted.

**Figure 10:**
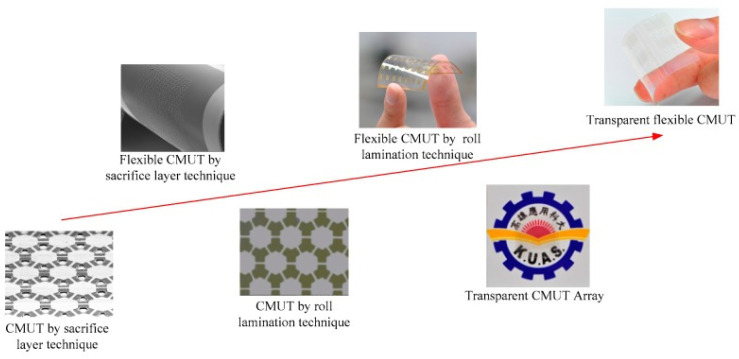
Flexible CMUT step process by Pang et al. [145].

Based on the advantages of CMUTs, a novel transparent flexible CMUT was developed using a roll-lamination technique with properties that demonstrated transparency, flexibility, and non-contact detection in display panels that offer a better human-to-machine interface (Figure 10) [145]. This CMUT was developed on a flexible substrate at a temperature below 100 °C to help reduce the stress of high temperatures. The developed CMUT is able to transmit pulse echoes, at various distances, which are then recorded given the frequency at which the echoes are transmitted.

Efforts have also been made to realize stretchable ultrasonic transducers. Most of the fabrication techniques for realizing stretchable ultrasonic transducers include using (1) a structural design for stretchable circuit patterning or a piezoelectric composite; (2) transfer printing; and (3) soft elastomeric packaging [146]. An ultrasonic probe with an island-bridge layout for biaxial stretchability was reported to have more than 50% stretchability, a high signal-to-noise ratio, a wide bandwidth, and a high spatial resolution to allow imaging through complex surfaces [147]. A similar device developed by Wang et al. to monitor the central blood pressure waveform was reported to be ultrathin, and to have stretchability of up to 60% with high sensitivity [146].

### 5.3. Wearable Antenna Communication

Wearable antennas are specifically designed to transmit and receive soft radio frequencies while being worn. This area of communication has been widened with the advent of wireless body area networks, which provide applications both within the medical and non-medical fields, such as medical sensing, body movement detection, skin monitoring, and other monitoring devices. Antennas help establish communication by linking the wearable device to a targeted device. Two possibilities exist for wearable devices: off the body and on the body. Off the body includes items such as bracelets, whereas on the body includes clothes, medical tape, and bandages. Some notable examples of antennas in various fields are fabric-based embroidered antennas, microfluidic antennas with injection alloys, and polymer-embedded antennas. Embroidered antennas include antennas such as Metal Composite Embroidery Yarn, Shieldex thread, carbon nanotubes, and Amberstrand fibers. Examples of microfluidic antennas are EGaIn and Galinstan. Zhibo Chen et al. showed the creation of a stretchable elastomer composed of Ag-PDMS for radio frequency passive components for various wireless wearable communication applications [148]. A wearable antenna is often the key component because it is responsible not only for receiving, but also transmitting, the signal used for the network of the device [149,150]. Table 4 provides details of the known frequency bands and the use of those frequencies in terms of applications [150].

### 5.4. Wireless Communication

**Figure 11:**
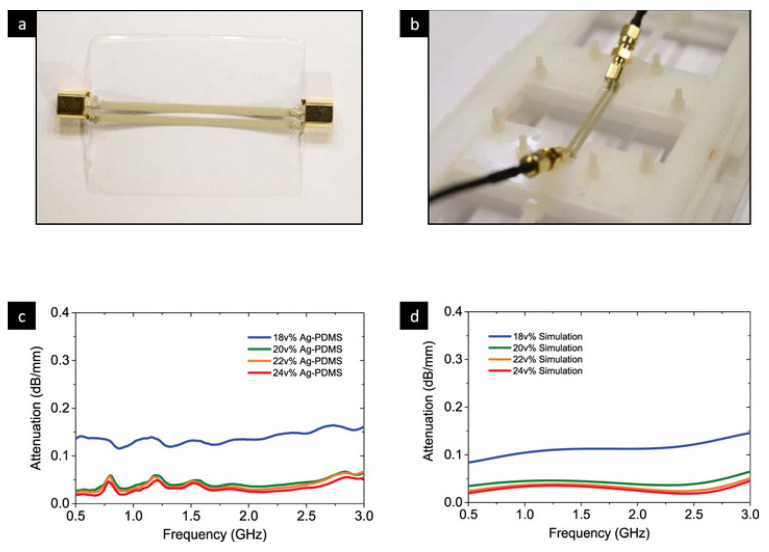
The Ag-PDMS sample with transmission line adapters seen in (**a**), (**b**) the tensile test fixture (**c**) graphing the different Ag volumes in comparison to the attenuation of transmission line, and (**d**) showing the attenuation of the transmission [148].

The use of wireless devices for communication and receiving information is not only limited to cellphones, but is now rising in significance within medical technology, from operating room equipment to home-healthcare devices that can be used for mobile phones. The use of interconnected wires limits the range of communication, in addition to posing an issue in regard to processing information and data collection. In the process of creating wireless forms of communication within biomedical devices, the general cost of creation and their efficiency of communication have been investigated. The devices are advancing from bulky models into soft deformable models that offer low cost and comfort for the user, combined with the freedom of not having to use bulky wires. A stretchable conductive elastomer was created that not only offers wireless communication, but also offers low-cost fabrication [148]. Zhibo Chen et al. designed a soft and deformable device with a stretchable conductor made from a mixture of Ag and PDMS (Figure 11). This mixture offered a high strain along with conductivity that enabled quality RF passive components for wireless communication. This device was designed to be mounted on the skin, with a flexible antenna having frequency ranges between 500 MHz and 3 GHz. The group’s data showed that the Ag-PDMS elastomer with a wireless form of communication was able to resist the signal loss of the RF passive front-end.

## 6. Power Sources

Throughout this article, we reviewed various flexible and stretchable electronics, their fabrication methods, and communication techniques. In addition, stretchable energy sources play an equally salient role in any kind of flexible and stretchable electronic device. Developing these energy sources presents some rewarding challenges. For an energy source to be used in flexible and stretchable electronics, several things must be considered, including the compliance of the material, the mechanical endurance under repetitive load, the amount of energy that it can provide to satisfy the application requirements, and the safety of the device. For continuous power generation and supply, researchers have explored the use of energy-harvesting components based on piezoelectric, triboelectric, photovoltaic, and thermoelectric mechanisms of power generation. Briefly, piezoelectric energy harvesters rely on the induced strain or pressure for electricity generation, as discussed previously. Triboelectric energy harvesters utilize the physical contact and separation between two dissimilar materials resulting from the biomechanical motion of the human body to generate electricity [151]. Photovoltaics cells have also been incorporated to convert light energy into electrical energy in self-powered ultra-flexible devices [152,153]. Similarly, thermoelectric energy generators have also been widely used in flexible devices to generate electrical power using the human body as the heat source [154,155]. Batteries and supercapacitors are the most widely used power sources in stretchable bioelectronics. Hence, we discuss these in further detail.

**Figure 12:**
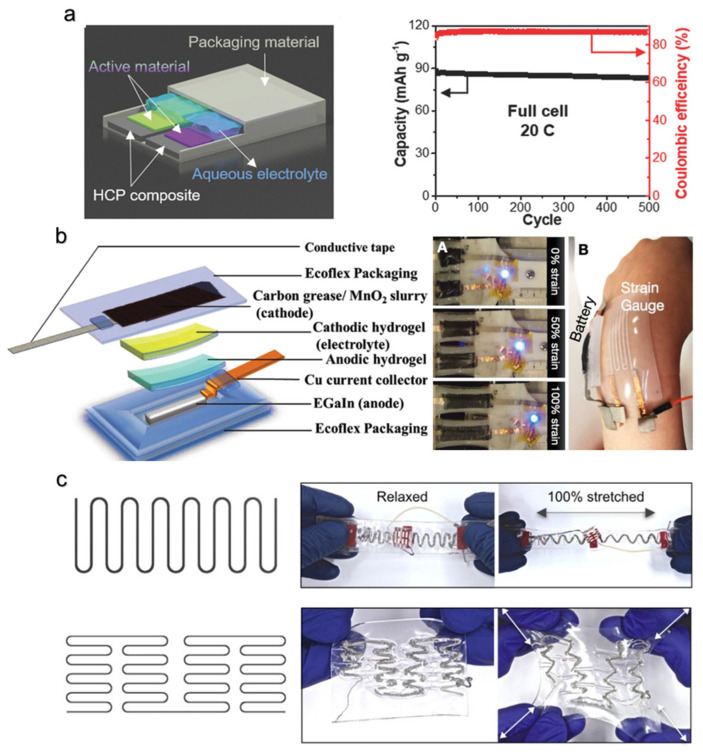
Stretchable batteries for powering bioelectronics. (**a**). Schematic showing a stretchable aqueous batteries configuration in which the HCP composite was used as a current collector. Long-term cycle performance and coulombic efficiency of the full cell at a rate of 20 C over 500 cycles [156]. Reprinted with permission from Ref. [156]. Copyright 2018 Wiley-VCH Verlag GmbH &Co. (**b**). Schematic showing the composition of a stretchable EGaIn battery [157] A. Photographs of the stretchable EGaIn-MnO_2_ battery array in series of two stretched under 0, 50, and 100% strain integrated with LEDs. B. Photograph of battery-powered strain sensor that is mounted on the wrist. Reprinted with permission from Ref. [157]. Copyright 2019 Wiley-VCH Verlag GmbH & Co. (**c**). Schematics of simple serpentine current collector and optical images of four serpentine-shaped batteries connected in series. Batteries continuously power an OLED while being subjected to a uniaxial strain of 100%. Schematics of a self-similar serpentine current collector and optical images of the full battery assembled around such a current collector. Geometry of the battery facilitates biaxial stretching [158].

Batteries store energy via an electrochemical process. Some batteries, such as Li-ion batteries, are widely used due to their design flexibility, ability to store large quantities of energy, and long-term cycling life [156]. The drawback of these batteries is they contain a non-aqueous electrolyte, from which arises the problem of flammability. When developing safe, stretchable batteries, one should also recognize that deformation may cause an internal short circuit, thus leading to explosion. By comparison, aqueous batteries demonstrate safety, high ionic conductivity, and cost effectiveness, and are therefore an ideal alternative to conventional batteries. An approach of incorporating hybrid carbon filler/polymer (HCP) composite as a current collector to fabricate aqueous Li-ion batteries was presented by the Song group (Figure 12a) [156]. They successfully demonstrated that such batteries provide a maximum power of 1260 W kg^−1^ and possess an excellent capacity retention of 93% after 500 charge–discharge cycles. Additionally, the HCP in these batteries is capable of straining up to 200%. Another similar effort was made by Liu’s team, in which they introduced liquid metal alloy, i.e., eGaIn-MnO_2_, rechargeable batteries for stretchable electronics [157]. The battery has a 3.76 mA h cm^−2^ specific areal capacity when fully discharged, exhibits stable discharge voltage within 100 cycles at 0.4 mA cm^−2^, and supports tensile strain of 100% (Figure 12b). In addition to employing the concept of aqueous batteries for stretchable bioelectronics, researchers have also implemented designs using helical spring-like and serpentine structures as a structural support and backbone for battery components. Zamarayeva et al. successfully implemented these designs to fabricate battery sources that have high specific capacity (~1.25 mA h cm^−1^) when charged at 0.25 C and discharged at 0.5 C [158]. Although the helical spring batteries showed that they can withstand flexing over 17,000 times to the bending radius of 0.5 cm, they found that these batteries cannot be stretched due to the mechanical properties of the polymer electrolyte, cellophane layer, and silver electrode used for the battery. The serpentine-shaped batteries overcame this issue because they can be readily stretched and can accommodate motions in a biaxial direction while retaining their electrochemical performance when stretched to 100% (Figure 12c).

**Figure 13:**
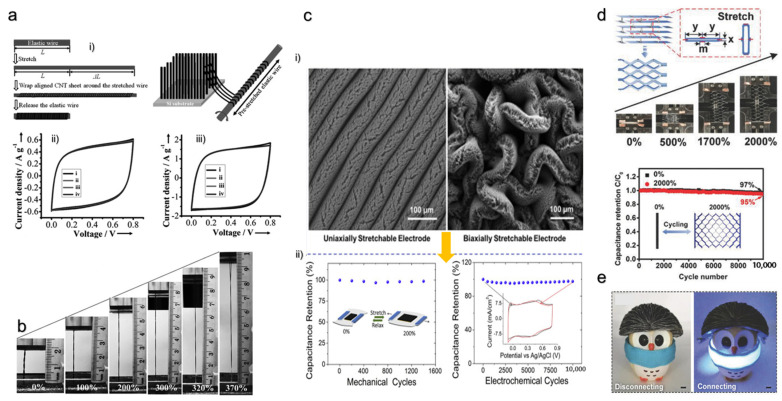
Supercapacitors for powering bioelectronic devices. (**a**) (i) Schematic illustration of fabricating stretchable conducting wire by wrapping an aligned CNT sheet around a pre-stretched elastic wire. CV curves of the supercapacitors based on (ii) the bare CNT-wrapped and (iii) CNT/PEDOT-PSS-wrapped wires [160]. (**b**) Digital photograph of a typical wire-shaped supercapacitor with a twisted structure after being stretched from strains of 0 to 370% [160]. Reprinted with permission from Ref. [160]. Copyright 2015 Wiley-VCH Verlag GmbH & Co. (**c**). Stretchable Au-CNT forest electrodes: (i) SEM image of the Au-CNT forest pattern morphology generated by a uniaxial pre-strain of 300% and by applying a biaxial pre-strain of 200% × 200%. (ii) Capacitance retention of a uniaxially stretchable Au-CNT forest electrode under mechanical stretching–relaxation cyclic deformations to a strain of 200% and for 10,000 charge/discharge cycles at the relaxed state. Inset shows the CV curves measured before and after the electrochemical stability test at the scan rate of 500 mV s^−1^ [159]. Reprinted with permission from Ref. [159]. Copyright 2020 Elsevier Inc. (**d**). Digital images of stretchable rectangular-shaped supercapacitors (with geometric parameters of y = 0.7 cm, m = 0.2 cm, x = 194 µm, T = 0.5 cm) under different strain tests. The inset images (upper left) are the scheme showing the expandable honeycomb structure and the hexagonal unit cell before and after being stretched. Capacitance retention ratio of 3D stretchable supercapacitor based on PPy/BPO-CNT electrodes tested at 7.8 mA cm^−2^ under the cycling tensile strain of 2000% [161]. (**e**). Arched bridge-shaped supercapacitors acting as a 3D helmet worn on the head of an owl toy model to power a 3.0 V flexible LED strip (right) [161]. Reprinted with permission from Ref. [161]. Copyright 2018 Wiley-VCH Verlag GmbH & Co.

Supercapacitors can survive millions more charging cycles than a rechargeable battery. However, they charge and discharge rapidly with massive bursts of energy while discharging. Generally, carbon nanomaterials such as CNTs and graphene have been explored for use in supercapacitors due to their low cost, high surface area, and long cycle lifetime [159]. Chen et al. demonstrated that highly stretchable supercapacitors with high electrochemical performance can easily be fabricated using a method involving the wrapping of CNT thin film synthesized by chemical vapor deposition around pre-stretched elastic wires, and twisting two of these CNT-wrapped elastic wires precoated with poly(vinyl alcohol) powder (PVA)/H_3_PO_4_/water hydrogel as the electrolyte (Figure 13a(i)) [160]. A PEDOT-PSS coating on CNT-wrapped elastic wires exhibited improved conductive and capacitive performance (Figure 13a(iii)). Supercapacitors based on CNT-wrapped wire showed excellent capacitance up to 30.7 F g^−1^ and high stretchability of up to a strain of 350%. Cao and Zhou’s team demonstrated a facile approach of fabricating crumpled CNT forest-based supercapacitors on a pre-strained elastomer substrate (Figure 13c) [159]. Such a crumpled CNT forest has the advantages of easy access due to its pore structure, short ion transport time, and low ionic diffusion resistance. With the deposition of Au on the CNT forest, they were able to demonstrate superior electrochemical performance (specific capacitance of ∼26 mF cm^−2^ for a biaxially stretchable Au-CNT forest) under a large deformation of 800% area strain regardless of charging and discharging rates.

Most of the supercapacitors fabricated on pre-stretched elastomers or textiles are 2D based. These supercapacitors have the limitations of the need for thin electrodes to achieve higher mechanical strain. This requirement prevents these supercapacitors from active material loading in the vertical direction, thereby resulting in low areal capacitance power. With this in mind, Lv et al. proposed honeycomb-lantern-inspired 3D supercapacitors (Figure 13d) [161]. They utilized the stretchability property of doped polypyrrole (PPy). To enhance stress relief and specific areal capacitance via effective ion transfer, they electrodeposited porous PPy and black phosphorus oxide (BPO) composites on CNT films. With this approach, they were successful in achieving supercapacitors that could maintain a capacitance ratio of 95% even when stretched at 2000% after 10,000 stretch and release cycles (Figure 13d), and had an enhanced specific areal capacitance of 7.34 F cm^−2^.

## 7. Conclusions

This review paper focused on various aspects required for the fabrication of a fully functional stretchable bioelectronic device. Therefore, it discussed several flexible and stretchable polymers, their mechanical and biocompatible properties, different strategies for making a device that is not only flexible but also stretchable, signaling and communicating methods, and stretchable power sources. The material section does not contain an exhaustive list of flexible and stretchable biomaterials. Those listed in Section 2 were only common biomaterials already deemed biocompatible with a multitude of applications. These materials are highly moldable. This area of research is moving fast and adapting. In fact, the use of organic materials such as proteins is a growing area for bioelectronics and offers better biocompatibility than polymers or metallic-based materials. Next, we discussed the common fabrication methods for manufacturing flexible and stretchable bioelectronic devices. The flexibility and stretchability of the device not only depends on the material properties of the substrate, but also on the mechanical properties of the electrodes or the conducting pathways to transmit the signal. Flexibility and stretchability in bioelectronic devices can be achieved using conductive polymer composites, the use of different structural configurations, such as serpentine geometry, wrinkled nanomembranes, or wavy structures, or the use of conductive liquids. Sensing the biochemical, biomechanical, or bioelectric cues of the human body, and communicating with an external data acquisition device, is an equally important aspect of the bioelectronic device. Piezoelectric devices, ultrasonic transducers, wearable antennas, and wireless forms of communication are a few of the trending methods of sensing and communication in the field of flexible and stretchable bioelectronics. Finally, we talked about the two major types of power sources (i.e., batteries and supercapacitors) that are widely used for stretchable bioelectronics.

Flexible/stretchable bioelectronics have attracted significant interest in health care applications as an alternative for bulky health-monitoring devices. Although the advances discussed above are noteworthy and provide a solid foundation for new classes of intelligent flexible/stretchable bioelectronics, several challenges remain to be addressed. Most of the implants or devices for chronic use are encapsulated by a polymer to create a barrier from biological fluids because these fluids can increase the rates of degradation and corrosion, or cause biofouling. This not only increases the thickness of the device and alters its mechanical properties, but also poses a challenge in the field of biosensing when monitoring the interstitial fluid [162]. Flexible and stretchable devices are an excellent choice when the device needs to be implanted on a flat surface, such as the skin or the heart, for constant monitoring of biosignals. However, what if the device has to be implanted on a rough surface or curvatures such as those of the brain or gastrointestinal (GI) tract? Due to this need for the conformability of the devices, the polymer or substrate must be conformable, so that the device adopts the curvature of the human body part where it is patched or implanted. Device conformability also ensures that the device is able to acquire changes in physiological cues or record bodily signals. As a result of the advance in highly stretchable and conformable substrates for a better interface with the human body arises the necessity of improved manufacturing technologies, such as low-temperature processes. As a hybrid approach or hard–soft material integration would lead to unwanted stress, localized strain, and hot spots in contact with human tissue [163], much effort is still needed in expanding the micro- and nano-fabrication techniques to accommodate the integration of hard semiconductors on soft, flexible, stretchable, and conformable devices. In addition to the available conductive inks/polymers, exploration of biocompatible and bioresorbable conductive inks/polymers is an active area of research. Furthermore, for most fabrication processes, crack formation has been observed in electrical conduits upon repeated cycles of straining the devices, and a tradeoff exists in mechanical and electrical properties in the case of using conductive polymer composites. These factors lead to a decrease in the sensitivity of the device; thus, research can be conducted to improve the device’s conductivity while maintaining its capability of sustaining high mechanical deformation. As batteries offer a high energy density and are an easy deployment option, efforts can be undertaken not only to reduce their size and increase their capacity [164], but also in creating batteries that are flexible and biodegradable [165]. Future flexible and stretchable bioelectronic devices can also be integrated with sensor arrays, on-site signal processing circuitry, and sensor calibration mechanisms for accurate signal analysis [166]. However, the integration of electronic components, especially power sources, into flexible/stretchable devices where polymer is often used as a substrate or a form of encapsulation has a disadvantage, namely that electronic components, including power sources, generate heat [167]. As the human body is sensitive to heat, efforts can be made to explore and develop a good thermally conductive material or a heat sink for bioelectronics [167]. If these challenges can be addressed, the next generation of bioelectronics will constitute a flexible, stretchable, and intelligent/automated multifunctional diagnostic and therapeutic bioelectronic system.

## Figures and Tables

**Table 1:** A table comparing the biocompatibility requirements (ISO 10993) for both implanted and wearable devices [32].

Wearable Devices	Implantable Devices
Nonirritating to Dermal tissue	Nonirritating, compliant to match surrounding tissue
Not cytotoxic, via leachable substances	Not cytotoxic
Either non degrading, or degraded substances are safe	Degraded substances are safe
Hemocompatibility (Indirect contact)	Hemocompatibility (direct contact)
	Minimize/eliminate biofouling

**Table 2:** Common bioelectronic materials with characteristics related to flexibility and stretchability. (all these values are for the listed applications, as each of the materials are highly tunable.).

Materials	Company	Young’s Modulus	Stretchability	Poisson’s Ratio	Applications
Silicone	Azo Materials	0.001–0.05 GPa	1000%–2000% [73]	0.47–0.49 [74]	Insulation
PDMS	Dow’s Sylgard 184	~1–3 MPa [75]	~1000% [47]	~0.5 [76]	Encapsulation/Nonconductive polymer
Polyimide	Dupont	2.5 GPa	260% [77]	0.34 at 23 °C	Neurocortical electrode arrays
Parylene-C	-	2.8 GPa, 3.2 GPa, 4.5 GPa [78,79]	20–200% [78]	0.4 [79]	Neural electrode substrate
PEDOT-PSS	-	1–7.5 GPa	~10% [80]	0.34	Conductive tracts
Polyurethane elastomer	-	4.7 MPa, 5.3 MPa, 7.4 MPa [48]	~1890% [51]	0.45–0.5	Vascular grafts, blood bags
Graphene	-	~1 TPa [81]	~30% [82]	0.456 ± 0.008 [83]	Logic gates, transistors

**Table 3:** Summary of fabrication methods with their advantages and disadvantages.

Fabrication Method	Advantages	Disadvantages
Chemical vapor deposition (CVD) Application: For creating thin films	Grow thin solid film on the substrate. Grow large-area and high-quality graphene film on metal substrate.	Need a metal (Ni, Cu) substrate to grow graphene layer. Cannot use polymer substrate to grow graphene layer. Challenge of growing large-grain crystal, which is required to enhance the electronic, mechanical, and thermal properties. Alternative: Modified CVD can be used to produce large-grain and highly crystalline film. Crystal growth variation at different areas. Requires transfer of the 2D film from the growth substrate to the target stretchable device substrate to add the stretchability property to the device. Possible contamination and damage during transfer of the film to the target.
Plasma-enhanced CVD (PECVD) Application: For creating thin films on polymer substrate.	Potential for direct growth of graphene layer on polymer substrate. Utilizes low processing temperature, providing an opportunity to use polymer as substrate.	Inferior graphene properties. Formation of cracks in the graphene structure may occur if repeatedly strained, leading to an increase in resistance.
UV lithography Application: For creating patterns for circuits for microelectronics, sensing, and optoelectronics. Can also be used to create high-aspect-ratio structures and 3D nanostructures such as nanowires (A novel top-down fabrication process for a vertically-stacked silicon-nanowire array).	Fabricate desired pattern ranging from nm to µm size on substrate. Soft and stretchable substrate can be used.	Can pattern only photoresist and may require a photomask for masked lithography [126,127]. Can be expensive if infrastructure such as a mask aligner is needed to develop complicated structures [128]. Resolution of the projection optics is diffraction-limited [126,129]. Challenging to design complex optics. Use of harsh processing conditions such as ion, plasma, acid, or temperature treatment to remove barrier layer, which limits the use of the substrate or photoresist that cannot handle such harsh processing [130].
Electron beam (E-beam) lithography Application: For producing patterns in a photomask	Produce a photomask. High-resolution approach to creating a photomask.	High resolution approach and therefore, time consuming. Not suitable for mass production.
Soft lithography Application: Thin film fabrication on soft substrates.	Micro/nano-structures can be fabricated on a soft substrate. Can be used to fabricate a mold. Easy, fast, low-cost, mass reproducible process. Does not require harsh processing chemicals.	Requires a stamp or a mold.
Printing Application: For creating thin film circuits of conductive liquids.	Can be used with conductive polymer composites and conductive liquid metals, thereby aiding in the fabrication of flexible and stretchable devices. Fast, accurate, and fully automated non-contact approach. Repeatable and scalable. Capability of achieving electronics with complex patterns without compromising the electrical conductivity of devices.	Requires good choice of conductive material and a solvent with appropriate viscosity, surface tension, and rheology. Requires the sintering process to create a conductive path.
Wet spinning method Application: For fabricating stretchable and electrically conductive fibers for wearable electronics (strain sensors, supercapacitors, nanogenerators).	Good technology for wearable electronics as the fibers can withstand mechanical deformations such as bending, folding, twisting, and stretching. A facile and viable strategy to fluidly spin the macroscopic fiber in a continuous way. Ability to control the diameter of the fiber by choosing an appropriate nozzle size of the spinneret.	Shear-thinning spinning ink is required for efficient flow. Requires selection of an appropriate coagulation bath so that the extruded spinning ink is rapidly vulcanized without any breakup.

**Table 4:** A wide variety of frequencies used when dealing with networks and antennas in wearable devices [150].

Frequency	Application
401–402 MHz	Medical Implant Communication Services (MICS).
402–405 MHz	
403.5–403.8 MHz	
405–406 MHz	
413–457 MHz	Medradio Micropower Networks (MMNs), transmit and relay data for implanted and body-worn medical devices for diagnostics and therapeutic functions.
2.4 and 5 GHz	Wi-Fi, smart hospital beds, mobile nursing stations.
2.404–2.478 GHz	Bluetooth, indoor navigation for patients, connectivity between the device and the smartphone for heath-data monitoring.
2.36–2.4 GHz	New Medical BAN (MBAN).
New Medical BAN (MBAN)	Industrial, Scientific, and Medical (ISM).
3.1–10.76 GHz	Ultra-Wideband (UWB).
57–64 G	The band plans and rules at mmW-60 BAN.
59–66 GHz	

## Data Availability

The study did not report any data.
